# Editorial Comment to CHARGE syndrome with both primary and secondary hypogonadism

**DOI:** 10.1002/iju5.12714

**Published:** 2024-02-25

**Authors:** Shoichiro Iwatsuki, Yukihiro Umemoto, Takahiro Yasui

**Affiliations:** ^1^ Department of Nephro‐urology Nagoya City University Graduate School of Medical Sciences Nagoya Japan; ^2^ Department of Urology Nagoya City University West Medical Center Nagoya Japan

CHARGE syndrome, which is a disorder with a combination of malformations, rarely occurs and, however, is an important cause of congenital hypogonadism. Most often dysfunction of chromodomain‐helicase‐DNA‐binding protein (CHD) 7 gene results in this disorder, causing multiple malformations and dysfunctions. CHD7 gene dysfunction impairs the function of the hypothalamus and pituitary gland which results in secondary hypogonadism.

Yoshida *et al*.^1^ reported a case of CHARGE syndrome with secondary but also primary hypogonadism that occurred in a 15‐year‐old boy. After stimulation with human chorionic gonadotropin (hCG), the patient's serum testosterone level did not elevate, which suggested the presence of mixed hypogonadism. The author decided to perform bilateral orchidectomy, and pathological examination revealed germ cell neoplasia in situ (GCNIS). Thus, the author hypothesized that^1^ dysfunction of the Leydig cell function was involved by decreased expression of steroid‐synthesizing enzymes and^2^ pathogenesis of GCNIS was involved in Sertoli cell degeneration associated with Leydig cell dysfunction.

CHD7 plays a role in the development of gonadotropin‐releasing hormone (GnRH) neuron. On the contrary, Hadziselimovic *et al*. reported a decrease in the expression of CHD7 in testicular tissue in boys with high infertility risk, which was defined by decreased spermatogonia.^2^ Additionally, overexpression of CHD7 in bladder cancer^3^ or mutation in the CHD7 gene in colorectal cancer^4^ has been reported, which suggests a hypothetical role of CHD7 on carcinogenesis. Thus, the direct effect of dysfunction of CHD7 in the testicular tissue should be considered. CHD7 is a member of the adenosine triphosphate (ATP)‐dependent helicase family.^5^ It regulates the expression of several genes by binding to histone or interacting with various transcription factors. To date, there has been no report mentioning the role of CHD7 in spermatogenesis or steroidogenesis. Further study is required to clarify the function of CHD7 in the testis.

However, this case report contributes toward clarifying the molecular mechanisms of spermatogenic failure and endocrinal dysfunction of the testis.

## Conflict of interest

The authors declare no conflict of interest.

## References

[1] Yoshida Y , Ogawa S , Meguro S *et al*. CHARGE syndrome with both primary and secondary hypogonadism. IJU Case Rep. 2024. 10.1002/iju5.12694 PMC11056257 38686072

[2] Hadziselimovic F , Gegenschatz‐Schmid K , Verkauskas G *et al*. Gene expression changes underlying idiopathic central hypogonadism in cryptorchidism with defective mini‐puberty. Sex. Dev. 2016; 10: 136–146. 27561106 10.1159/000447762

[3] Ware AP , Satyamoorthy K , Paul B . Integrated multiomics analysis of chromosome 19 miRNA cluster in bladder cancer. Funct. Integr. Genomics 2023; 23: 266. 37542643 10.1007/s10142-023-01191-0 PMC10404189

[4] Tahara T , Yamamoto E , Madireddi P *et al*. Colorectal carcinomas with CpG Island methylator phenotype 1 frequently contain mutations in chromatin regulators. Gastroenterology 2014; 146: 530–538. 24211491 10.1053/j.gastro.2013.10.060 PMC3918446

[5] Bouazoune K , Kingston RE . Chromatin remodeling by the CHD7 protein is impaired by mutations that cause human developmental disorders. Proc. Natl. Acad. Sci. USA 2012; 109: 19238–19243. 23134727 10.1073/pnas.1213825109 PMC3511097

